# Single-cell and Bulk RNA-Seq reveal angiogenic heterogeneity and microenvironmental features to evaluate prognosis and therapeutic response in lung adenocarcinoma

**DOI:** 10.3389/fimmu.2024.1352893

**Published:** 2024-02-08

**Authors:** Lijuan Tang, Zhike Chen, Jian Yang, Qifan Li, Sichu Wang, Taoming Mo, Weibiao Zeng, Hao Ding, Shu Pan

**Affiliations:** ^1^ Dalian Medical University, Dalian, China; ^2^ Department of Pathology, Affiliated Hospital of Nantong University, Nantong, China; ^3^ Department of Thoracic Surgery, The First Affiliated Hospital of Soochow University, Suzhou, China; ^4^ Medical School of Nantong University, Nantong, China; ^5^ Suzhou Gene Pharma Co., Ltd, Suzhou, China

**Keywords:** angiogenesis, tumor microenvironment, immune infiltration, immune therapy, prognosis, lung adenocarcinoma

## Abstract

**Background:**

Angiogenesis stands as a pivotal hallmark in lung adenocarcinoma (LUAD), intricately shaping the tumor microenvironment (TME) and influencing LUAD progression. It emerges as a promising therapeutic target for LUAD, affecting patients’ prognosis. However, its role in TME, LUAD prognosis, and its clinical applicability remain shrouded in mystery.

**Methods:**

We employed integrated single-cell and bulk transcriptome sequencing to unravel the heterogeneity of angiogenesis within LUAD cells. Through “consensus clustering”, we delineated distinct angiogenic clusters and deciphered their TME features. “Monocle2” was used to unravel divergent trajectories within malignant cell subpopulations of LUAD. Additionally, regulon submodules and specific cellular communication patterns of cells in different angiogenic states were analyzed by “pyscenic” and “Cellchat” algorithms. The “univariate Cox” and “LASSO” algorithms were applied to build angiogenic prognostic models. Immunohistochemistry (IHC) on clinical samples validated the role of model factors in LUAD angiogenesis. We utilized CTRP 2.0 and PRISM databases for pinpointing sensitive drugs against lung adenocarcinoma.

**Results:**

Two clusters for the activation of angiogenesis were identified, with Cluster 1 showing a poor prognosis and a pro-cancerous TME. Three differentiated states of malignant epithelial LUAD cells were identified, which had different degrees of angiogenic activation, were regulated by three different regulon submodules, and had completely different crosstalk from other cells in TME. The experiments validate that SLC2A1 promotes angiogenesis in LUAD. ARS (Angiogenesis related score) had a high prognostic value; low ARSs showed immunotherapy benefits, whereas high ARSs were sensitive to 15 chemotherapeutic agents.

**Conclusion:**

The assessment of angiogenic clusters helps to determine the prognostic and TME characteristics of LUAD. Angiogenic prognostic models can be used to assess the prognosis, immunotherapeutic response, and chemotherapeutic drug sensitivity of LUAD.

## 1 Introduction

Lung cancer is the most common cause of cancer-related death (1), and lung adenocarcinoma (LUAD) is its leading pathological type (2), which accounts for 50% of all lung cancer cases (3). Tumor heterogeneity is the main cause of drug resistance and tumor recurrence in LUAD (4), and the complex tumor microenvironment (TME) is key to LUAD heterogeneity (5). Chemotherapeutic and immunotherapeutic efficacy exhibit varying degrees of heterogeneity in patients with LUAD (6), thus hindering precise assessment of individual patient prognosis. Recent studies have suggested that the components of TME can determine the cancer immunophenotype and help guide chemotherapy and immunotherapy stratification in the future (6–8).

Angiogenesis is defined as the formation of new blood vessels from pre-existing vessels through a process called germination. Angiogenesis is important for the phenotypic differentiation of TME (9). Vascular endothelial growth factor (VEGF) is a critical driver of tumor neo-angiogenesis, and its expression within TME is heterogeneous, leading to an immunosuppressive effect (10). VEGFA exerts angiogenic effects by activating VEGFR2 expressed on endothelial cells (11). In recent years, anti-angiogenic drugs targeting the VEGFA pathway have significantly contributed to the treatment of LUAD (12).

Cancer-associated fibroblasts within TME are involved in angiogenesis, immune escape, and drug resistance (13). Tumor-associated macrophages (TAMs) are enriched in TME in most cancer types. TAMs polarise into the M1 or M2 phenotype depending on the environment, and M2 macrophages express anti-inflammatory cytokines (e.g. IL-10, CCL22, and CCL18) and low levels of IL-12, thereby exerting anti-inflammatory, angiogenic and pro-tumor effects (14). Chemokines in TME mediate the recruitment of immune cells to TME and directly affect cancer and endothelial cells to regulate tumor neo-angiogenesis (15). Furthermore, angiogenesis modulates metabolism and immunity. An abnormal vascular system inevitably leads to hypoxia and acidosis, resulting in the upregulation of tumor factors such as VEGF and TGF-β in the TME and eventually promoting metastasis and immunosuppression (16). Therefore, the regulation of angiogenesis is extremely complex and closely related to the TME. However, no multi-omics study of LUAD based on angiogenesis-related genes has analyzed their specific role in the TME and prognosis.

Employing scRNA-seq, we can analyze RNA profile variations at a high resolution to comprehend the intricate tumor microenvironment (TME) (17). Previous LUAD studies utilized scRNA-seq to explore diverse cell profiles within the microenvironment. In this study, distinct angiogenic clusters were identified based on 36 previously reported angiogenesis-related genes. We revealed heterogeneity of angiogenic activity in the LUAD tumor microenvironment at the single-cell level. Additionally, to enhance clinical applicability, an angiogenic scoring system was developed. This system evaluates LUAD aggressiveness and TME phenotype, guiding the customization of chemotherapy and immunotherapy strategies for individualized patient care.

## 2 Materials and methods

### 2.1 Pre-processing of bulk RNA-seq data

The gene expression data and clinical information of patients with LUAD were downloaded from the NCBI GEO (https://www.ncbi.nlm.nih.gov/geo/) and TCGA (https://cancergenome.nih.gov/) databases. A total of 884 LUAD samples from the GSE31210 (N = 226), GSE42127 (N = 133), GSE50081 (N = 127), and GSE72094 (N = 398) datasets were included in this study. The RNA-seq data (FPKM format, N = 500) and survival information of patients with LUAD were extracted from the TCGA database and converted to the transcripts per million (TPM) format. The Combat algorithm of the R package “SVA” was used to remove batch effects in samples from the GEO datasets. All data were log2(X+1) normalized for subsequent analysis. The somatic gene mutation data of patients with LUAD were downloaded from the UCSC Xena database (https://xenabrowser.net/datapages/).

### 2.2 Extraction and manipulation of single-cell RNA-seq data

Raw scRNA-seq data were downloaded from the GSE127465 dataset for single-cell analysis. The data contains 12 samples from 5 lung adenocarcinoma patients. In addition, the expression matrix, cell clustering, and cell type annotation data of the dataset were downloaded from the TISCH database (17). Samples with UMI counts of >1000 and >500 genes expressed in each cell were retained. For subsequent analysis of malignant epithelial LUAD cells, the number of highly variable genes was set to 2000, and the resolution was set to 0.6 for cell clustering. The data were dimensionalized using the “tSNE” method, and differentially expressed genes among malignant cell clusters were calculated using the “FindAllMarkers” algorithm.

### 2.3 Consensus clustering of angiogenic clusters

We extracted a set of 36 angiogenesis-related genes from MsigDB (http://www.gsea-msigdb.org/gsea/msigdb/search.jsp) for this study. Utilizing the R package “ConsensusClusterPlus”, we conducted consensus clustering analysis on the gene expressions. The algorithm employed was “KM”, using “euclidean” distance calculation and a random seed set to “5555555”. The GEO and TCGA-LUAD cohorts were categorized into two expression patterns, Cluster1 and Cluster2. Differential gene expression between the clusters was identified using the R package “limma”.

### 2.4 ssGSEA, GSVA, and single-cell functional gene set activity scores

Transcriptomic pathway activity scores were assessed using gene set variation analysis (GSVA) with the “HALLMARK dataset”. Enrichment scores were calculated using single-sample gene set enrichment analysis (ssGSEA) to represent the activity scores of cancer-related biological pathways and immune microenvironment-related signatures. Functional activity scores for each cell were determined using the “SingleCellSignatureScorer” software, relying on the differential expression of genes between the two expression clusters (18).

### 2.5 GO and KEGG enrichment analyses and GSEA

GO and KEGG functional enrichment analyses of differentially expressed genes were performed using the R package “clusterProfiler”. GO analysis included functional enrichment of biological processes (BP), cellular components (CC), molecular functions (MF), and other categories.

### 2.6 Single-cell trajectory analysis

Based on the single-cell data (Seurat objects), single-cell trajectories were constructed using the R package “Monocle2”, and genes regulated in a branch-dependent manner were identified using the branched expression analysis modeling (BEAM) algorithm (19).

### 2.7 Cell communication analysis

Based on the human CellChatDB database, cellular communication among LUAD cells of different trajectory branches, immune, and stromal cells in TME was analyzed using the R package “CellChat”. In addition, ligand–receptor pairs involved in different signaling pathways in tumor, immune, and stromal cells were identified.

### 2.8 Identification of Regulon submodules

A list of human transcription factors was downloaded from the RcisTarget database (https://resources.aertslab.org/cistarget/) and used to construct a transcription factor regulatory network. The “pyscenic” algorithm in Python was used to build a gene co-expression network based on the abovementioned transcription factors, establish transcription factor–target regulatory relationships, and identify a regulon (20). In addition, the regulon activity score (RAS) of cells was evaluated using the “AUCell” algorithm. The area under the curve (AUC) and connection specificity index (CSI) were calculated, and the regulon submodules were defined by hierarchical clustering of regulons based on CSI.

### 2.9 Immunohistochemistry

A total of 18 lung adenocarcinoma samples, along with 7 corresponding paracancerous tissues, were collected. Ethical approval has been obtained from the Medical Ethics Committee at The Affiliated First Hospital of Soochow University for the collection of tissue specimens. The tissues were fixed with 4% paraformaldehyde, dehydrated, and paraffin-embedded, resulting in 4 μm sections. Tissue sections underwent incubation at 4°C overnight with primary antibodies targeting SLC2A1 (Sangon, D160433, 1:200), CD34 (Sangon, D363155, 1:200), and VEGFA (Sangon, D260788, 1:200) post-deparaffinization, rehydration, and antigen retrieval. Subsequently, the slides were exposed to an antirabbit secondary antibody, followed by DAB staining and hematoxylin counterstaining. Two blinded pathologists independently assessed the immunohistochemistry (IHC) results. Tissue sections were scored based on the percentage of positive cells and staining intensity. Staining intensity was graded as 0 (negative), 1 (weak), 2 (moderate), or 3 (strong), while the expression proportion of positive cells was scored as 1 (0–25%), 2 (26–50%), 3 (51–75%), or 4 (76–100%). The proportion and intensity scores were amalgamated to derive a final score. An IHC score of ≥6 denoted high expression, while <6 indicated low expression.

### 2.10 Analysis of immunotherapy response and chemotherapy drug sensitivity

Data regarding the response of patients with LUAD to immunotherapy were extracted from the GSE126044 (N = 16) cohort, and immunotherapeutic efficacy was predicted using the TIDE algorithm (http://tide.dfci.harvard.edu/). Data regarding the sensitivity of patients to chemotherapeutic drugs were extracted from the CTRP 2.0 database (https://portals.broadinstitute.org/ctrp.v2.1/), and AUC data for PRISM analysis were extracted from the PRISM Repurposing Secondary Screen 19Q4 dataset (https://depmap.org/portal/download/). The area under the dose-response curve (AUC) in both datasets was used to measure drug sensitivity, with lower AUC values indicating higher sensitivity. Differences in drug sensitivity were analyzed using the Wilcoxon test and Spearman correlation analysis (log2FC > 0.15, r < –0.4). Missing AUC values in the dataset were imputed using the K-nearest neighbors (KNN) algorithm, and chemotherapeutic drugs with >20% missing data were excluded (20). The expression profile data of the CCLE cell line (https://portals.broadinstitute.org/ccle/data) were used as a training set for predicting drug sensitivity. Drug response in each sample was evaluated using the pRRophetic package.

### 2.11 Statistical analysis

Statistical analyses were performed using the R software (version 4.2). For comparing the data of two datasets, the significance of normally distributed variables was estimated using the Student t-test, whereas that of non-normally distributed variables was estimated using the Wilcoxon test. For comparing the data of more than two groups, one-way ANOVA was used to analyze normally distributed data, whereas the Kruskal–Wallis test was used to analyze non-normally distributed data. The two-sided Fisher exact test was used for R*C tables containing <5 samples. Kaplan–Meier survival analysis and Cox proportional hazards model were used to analyze the significance of prognostic features. A multivariate regression model was used to adjust for confounders. The Benjamini–Hochberg method was used to control the false discovery rate (FDR) for multiple hypothesis testing, with all comparisons being two-sided with an alpha level of 0.05 (21) (*, P < 0.05; **, P < 0.01; ***, P < 0.001).

## 3 Results

**Figure 1:**
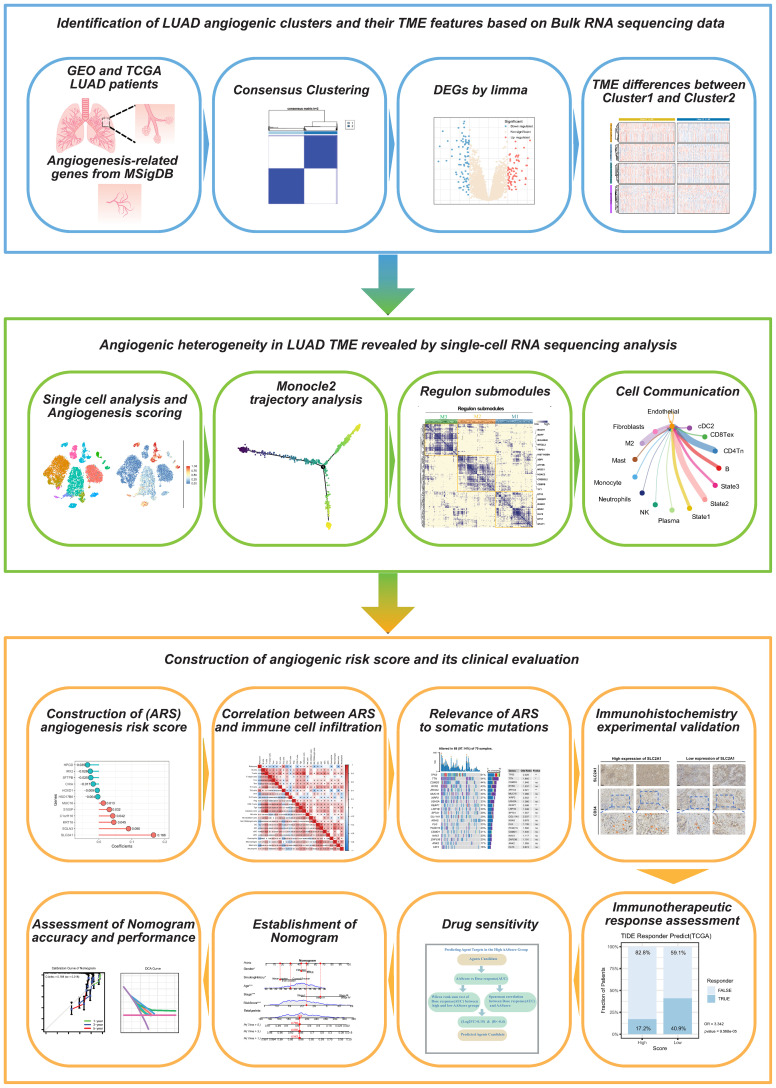
The flow chart of this study.

**Figure 1** shows the flow chart of this study.

### 3.1 Identification of angiogenic clusters for LUAD

**Figure 2:**
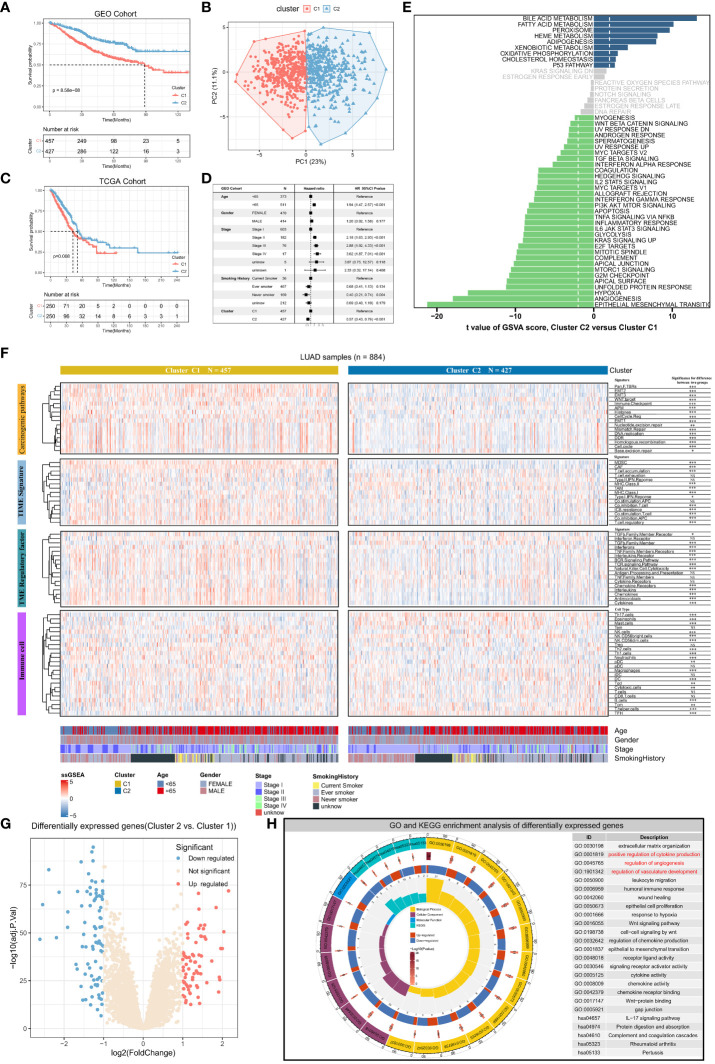
Angiogenic clusters distinguish tumor microenvironment phenotypes and prognostic characteristics in lung adenocarcinoma. **(A)** Kaplan-Meier curves for overall survival (OS) of lung adenocarcinoma patients with different angiogenic cluster in the GEO cohort, Log-rank test P<0.001. **(B)** Principal component analysis based on 36 genes related to angiogenesis can well distinguish the two angiogenic clusters. **(C)** Overall survival (OS) Kaplan-Meier curves for lung adenocarcinoma patients in the TCGA cohort with different angiogenic cluster, Log-rank test P=0.008. **(D)** Multivariate Cox regression analysis based on clinicopathological characteristics of patients to assess the prognostic value of angiogenic cluster in lung adenocarcinoma. **(E)** Enrichment scores for the 50 “Hallmark “ gene sets in lung adenocarcinoma patients were assessed using the GSVA algorithm and tested for the significance of differences, with the horizontal axis indicating the t-value of the difference analysis. Entries with |t value| > 1.96 in this study were statistically significant, and a negative t value indicated that the signaling pathway was actively expressed in Cluster1. **(F)** The enrichment scores of Carcinogenic pathways, TME signature, TME regulatory factor, and immune cell signatures were evaluated based on the ssGSEA algorithm, and displayed with Heatmap and compared the difference in enrichment scores between the two angiogenic clusters. **(G)** Significantly differentially expressed genes (DEGs) between the two angiogenic clusters, 72 genes were upregulated and 81 genes were downregulated in Cluster2. **(H)** Functional annotation of DEGs using GO and KEGG functional enrichment analysis. The innermost circle represents the number of enriched genes in the corresponding pathway, and the remaining circle meanings have been labeled in the center of the circle.

We conducted consensus clustering analysis on lung adenocarcinoma patients using expression data of 36 angiogenesis-related genes to differentiate angiogenic clusters of LUAD. Two clusters, namely, Cluster1 and Cluster2, were identified using LUAD samples in the GEO dataset (**Supplementary Figures S1A–C**). The two clusters possess different angiogenic gene expression patterns and are associated with different prognoses, with Cluster 1 having a worse prognosis (P < 0.001, log-rank test) (**Figure 2A**). Principal component analysis revealed that the two clusters were completely distinguishable based on the expression of angiogenesis-related genes (**Figure 2B**). Samples from both clusters were evenly distributed in the independent GEO cohort, and only Cluster 1 showed a worse prognosis (**Supplementary Figures S1D–G**). Consensus clustering was performed in the TCGA-LUAD cohort using the same method (**Supplementary Figures S1H, I**), and similar results were obtained (**Figure 2C**). The results of multivariate Cox analysis validated that the angiogenic clusters identified based on angiogenesis-related genes might serve as independent prognostic factors for LUAD (Cluster2 versus Cluster1; HR, 0.57; 95% CI, 0.43–0.76; P < 0.001) (**Figure 2D**). Next, the GSVA algorithm evaluated Hallmark gene sets to explore potential biological mechanisms of the differences between the two clusters. Cluster1 was significantly enriched in various oncogenic pathways, such as TGF-β signaling, epithelial–mesenchymal transition, angiogenesis, hypoxia, and apoptosis, whereas Cluster2 was mainly involved in the activation of biological pathways, such as the P53 signaling pathway and fatty acid metabolism (**Figure 2E**). These results suggest that angiogenesis is closely related to the TME of LUAD and is involved in LUAD development.

### 3.2 Differences in TME characteristics between angiogenic clusters

To understand the tumor microenvironmental phenotype mapped by angiogenic clusters, the activity of signatures associated with cancer-related pathways was analyzed using the ssGSEA algorithm. The results indicated that the expression of multiple signatures was significantly different between the two clusters. The expression of signature genes associated with cancer-related pathways including EMT, WNT targeting, cell cycle, antigen presentation, and immune checkpoints was higher in Cluster1 than in Cluster2 (P < 0.001) (**Figure 2F**). Furthermore, differences in immune and stromal cell regulation between the two clusters were analyzed. Stromal cells with pro-oncogenic effects (e.g., MDSCs and CAFs) and regulatory T cells that suppress anti-tumor immunity were more active in Cluster1. Meanwhile, the expression of genes associated with immune checkpoint blockade (ICB) resistance was also high in Cluster1. However, despite the aggregation of various cancer-promoting stromal and immune cells in Cluster1, MHC and co-stimulatory molecules were activated, suggesting that anti-cancer immune responses are also related to Cluster1. These results indicate that immune cells and pro-cancer biological pathways play an important role in Cluster 1. Besides, there are complex chemokine and cytokine regulatory networks in TME, and we found that there are entirely different regulatory factor expression levels for different angiogenic expression patterns based on the ssGSEA enrichment results of the signature of these tumor microenvironmental regulators. For example, BCR (B cell receptor) signaling, TCR (T cell receptor) signaling, natural killer cell cytotoxicity, interleukin expression, chemokine expression, and cytokine expression were significantly upregulated in Cluster1, suggesting that the destabilization of chemokine and cytokine regulation in Cluster1 leads to a poor prognosis of LUAD. Furthermore, immune cell infiltration was analyzed in the two clusters. The infiltration of T helper, TFH (Follicular helper T cell), DC (Dendritic cells), mast, Tem (Effective Memory T Cell), and Th17 cells was significantly high in Cluster2, whereas that of macrophages and neutrophils was significantly high in Cluster1. These results validated our previous hypothesis, indicating that the pro-oncogenic immune microenvironment and pathways predominated in Cluster1, which suggests that elevated angiogenic activity accompanies the pro-oncogenic TME.

The two angiogenic clusters exhibited distinct tumor microenvironmental phenotypes. Differentially expressed genes (|log2fold change| > 1, adj. P < 0.05) between angiogenic Cluster1 and Cluster2 were identified as angiogenic clusters-related genes (**Figure 2G**). Subsequent GO and KEGG functional enrichment analysis revealed significant enrichment in the extracellular matrix, cytokine and chemokine production, angiogenesis regulation, immune response regulation, Wnt signaling pathway, and EMT-related processes. This validates that the differentially expressed genes exhibit characteristics of angiogenesis and its mediated TME (**Figure 2H**), reflecting differences in angiogenic clusters and their underlying biological mechanisms.

### 3.3 Angiogenic heterogeneity among different cell types and subtypes

**Figure 3:**
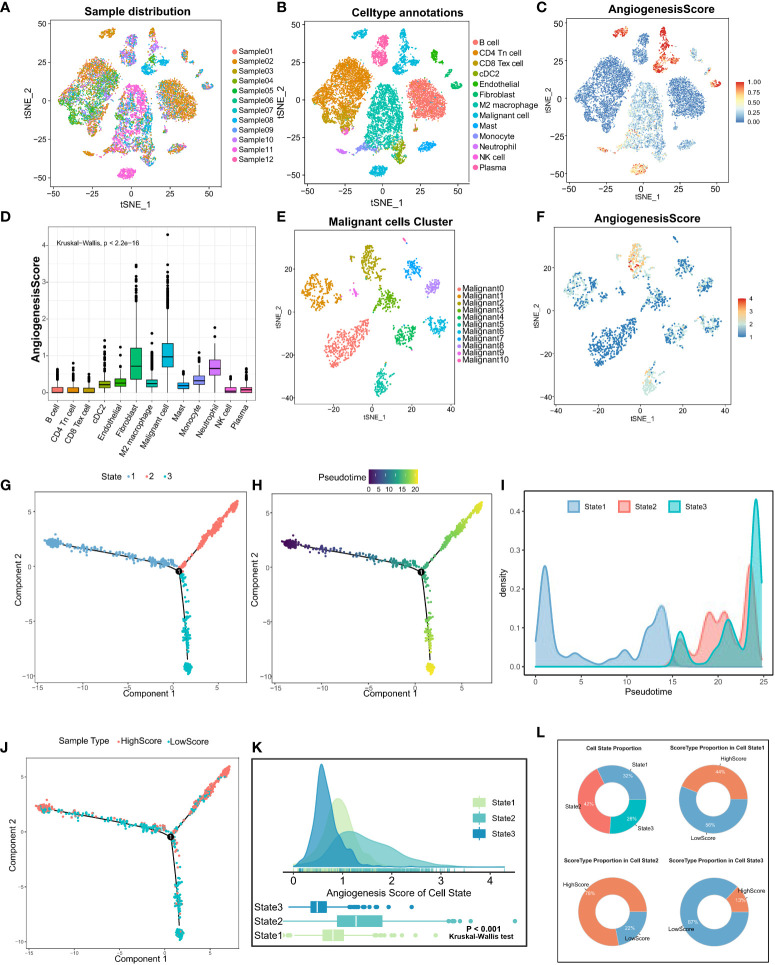
Analysis of angiogenic scores at the cellular level and trajectory analysis by single-cell sequencing. **(A)** The integration effect of 12 samples of lung adenocarcinoma samples appeared to be good with no significant batch effect. **(B)** Reduced-dimension visualization of tSNE of lung adenocarcinoma cells, each color represents a cluster, and the cell type represented by each color is labeled on the right. **(C)** Angiogenesis scores of cells were assessed based on DEGs between angiogenesis clusters. **(D)** The Kruskal-Wallis test for heterogeneity of angiogenesis scores between different cell types. **(E)** Reduced dimensional clustering of tSNE of malignant cells in lung adenocarcinoma, each color represents a cluster, and the cell type represented by each color is labeled on the right. **(F)** Visualization of angiogenesis score of Malignant cells in lung adenocarcinoma. Pseudo time analysis of Malignant cells based on Monocle2 inference, **(G)** each color represents one cell State, **(H)** shows pseudo time analysis changes and pseudo time start and endpoints. **(I)** Density diagram showing the process of cell State changes with pseudo-time. **(J)** The mapping of pseudo time distribution to high and low angiogenesis scores. **(K)** Kruskal-Wallis test for comparing significant differences in angiogenesis scores between the three cell State states. **(L)** State type proportion statistics of Malignant cells in lung adenocarcinoma and the proportion composition of HighScore and LowScore groups of different cell States were counted separately.

To explore the heterogeneity of angiogenic activity among cell types, angiogenic clusters-related genes were used as the angiogenic signature, and scored using the “SingleCellSignatureScorer” algorithm. Firstly, a total of 12 samples in the scRNA-seq dataset had a good integration effect among samples, with no significant batch effect, thus allowing for subsequent analysis (**Figure 3A**). Through descending and unsupervised clustering, samples were classified into 13 cell types, encompassing immune, stromal, and malignant tumor cells (**Figure 3B**). Angiogenesis scores, reflecting the degree of biological activity, varied among these cell types. Notably, fibroblasts, malignant cells, and neutrophils displayed significantly higher scores than immune cells, indicating more active angiogenesis (**Figures 3C, D**).

Furthermore, focusing on the heterogeneity of scores among malignant tumor cells, the cells were divided into 11 different subtypes (**Figure 3E**). Similarly, significant differences in angiogenesis scores were observed in different subpopulations of malignant tumor cells (**Figure 3F**). Altogether, these results suggest that different cells in TME exhibit different levels of angiogenesis. Therefore, it is important to investigate the causes of angiogenic dysregulation.

To examine the important role of angiogenesis in malignant cell heterogeneity, cellular pseudo-time analysis was performed to investigate malignant cell differentiation trajectories. The results revealed three main differentiation states of malignant cells, namely, State1, State2, and State3 (**Figure 3G**). Malignant cells in State1 are the initiating factors of the reverse chronological trajectory, whereas State2 is at the end of the trajectory. (**Figure 3H**). The transition of State1, State2, and State3 with pseudotime can be visualized clearly through density diagrams and trajectory plots. (**Figures 3I, J**). Furthermore, significant differences in angiogenesis scores were observed among the three cell states (Kruskal–Wallis test; P < 0.001) (**Figure 3K**). State3 had the lowest angiogenesis scores (low-score group), and State2 had the highest scores (high-score group) (**Figure 3L**), suggesting that angiogenesis is involved in malignant cell heterogeneity. In addition, angiogenesis is dysregulated in LUAD, and its activation is closely related to the differentiation status of LUAD cells.

### 3.4 Regulon submodules of different cell states

**Figure 4:**
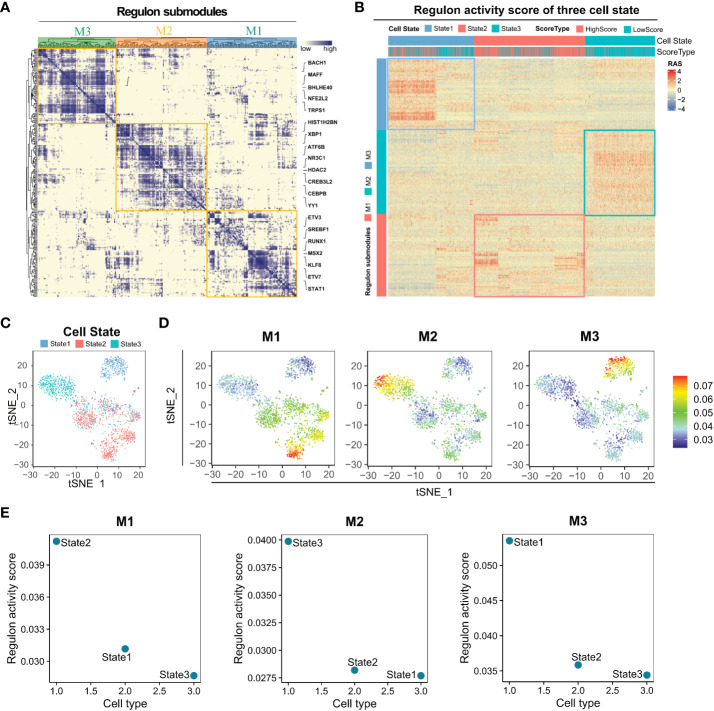
Distinct regulon submodules activation in State1, State2, and State3 cells. **(A)** The transcription factors of different States of lung adenocarcinoma Malignant cells can be clustered into three regulon submodules, M1, M2, and M3. **(B)** Regulon activity score for regulon submodules in three cell states. **(C)** Visualization of the tSNE reduced the dimensionality of three cell States. **(D)** The Regulon activity score has been mapped to each cell. **(E)** Regulon activity scores of M1, M2, and M3 regulon submodules in three cell states.

Clustering regulons based on the Connection Specialty Index (CSI) revealed three submodules, M1, M2, and M3 (**Figure 4A**). Regulons within the same submodule exhibited tight expression correlations. Subsequently, regulon activity scores were calculated for the three cell states, indicating the activation of regulons in each state. M1, M2, and M3 module regulons were predominantly activated in State2, State3, and State1, respectively (**Figures 4B–D**). The M1 module regulon, associated with high angiogenic scores, appeared to primarily regulate angiogenic activation (**Figure 4E**). The establishment of a regulon-based regulatory network enhances our understanding of the three cell differentiation states and aids in identifying markers and therapeutic targets for LUAD.

### 3.5 Cell communication of malignant cells with TME

**Figure 5:**
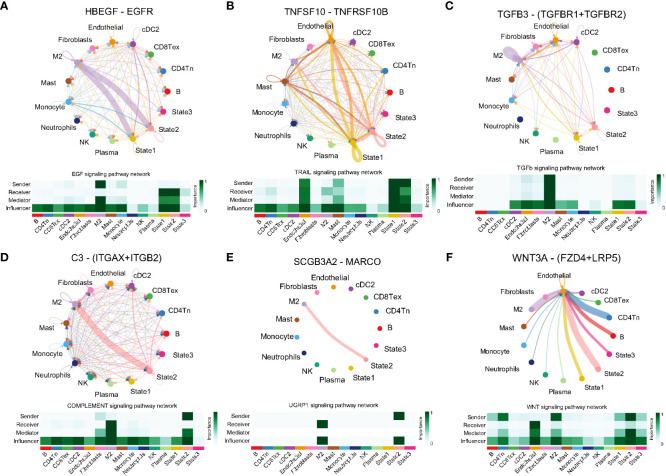
Ligand receptor pairs mediating cell communication between cell state2 and the tumor microenvironment. **(A)** State2 cells communicate with M2-type macrophages via HBEGF-EGFR. **(B)** State2 cells in concert with State1 communicate closely with Mast and Endothelial via TNFSF10-TNFRSF10B. **(C)** State2 cells communicate with State1 synergistically via TGFB3-(TGFBR1+TFGBR2), **(D)** C3-(ITGAX+ITGB2), **(E)** SCGB3A2-MARCO and M2 macrophages. **(F)** State2 intercommunicates with Endothelial via WNT3A-(FZD+LRP5).

The findings indicate an association between angiogenesis and the microenvironment of lung adenocarcinoma. Cell communication pattern recognition predicts how cells, as signal senders or receivers, coordinate with each other and signaling pathways to drive intercellular communication. In this study, we analyzed cell communication within the lung adenocarcinoma TME involving malignant cells, immune cells, and stromal cells. The results revealed there were two incoming signal coordination modes and two outgoing signal coordination modes for intercellular communication and the signaling pathways coordinated with it (**Supplementary Figure S2A**). State1 cells can be signalled via the TWEAK signalling pathway (TNFSF12–TNFRSF12A, **Supplementary Figure S2B**), IGF signalling pathway (IGF2–[ITGA6+ITGB4], **Supplementary Figure S2C**), MK signalling pathway (MDK–[ITGA6+ITGB1], **Supplementary Figure S2D**), SEMA3 signalling pathway (SEMA3B–[NRP2+PLXNA2], **Supplementary Figure S2E**) and PERIOSTIN signalling pathway (POSTN–[ITGAV+ITGB5], **Supplementary Figure S2F**) for active communication with M2 macrophages, endothelial cells, and CD4 T cells. State2 cells can be signaled through the EGF signaling pathway (HBEGF–EGFR, **Figure 5A**), TRAIL signaling pathway (TNFSF10–TNFRSF10B, **Figure 5B**), TGF-β signaling pathway (TGFB3–[TGFBR1+TGF, **Figure 5C**), complement signaling pathway (C3–[ITGAX+ITGB2], **Figure 5D**), UGRP1 signaling pathway (SCGB3A2–MARCO, **Figure 5E**) and WNT signaling pathway (WNT3A–[FZD4+LRP5], **Figure 5F**) for active communication with M2 macrophages, mast cells, and endothelial cells. It is interesting to note that there are similar results between State1 and State2 cells. However, State3 cells communicate closely with M2 macrophages, fibroblasts, endothelial cells, and cDC cells through a signaling pathway that is distinct from that associated with State1 and State2 cells (**Supplementary Figure S3**). Although the cell types that communicate with cells in the three states are similar, the signaling pathways are different, indicating that heterogeneity of the angiogenic regulatory microenvironment is closely related to these signaling pathways.

### 3.6 Construction of the angiogenic risk score and discussion of its clinical relevance

**Figure 6:**
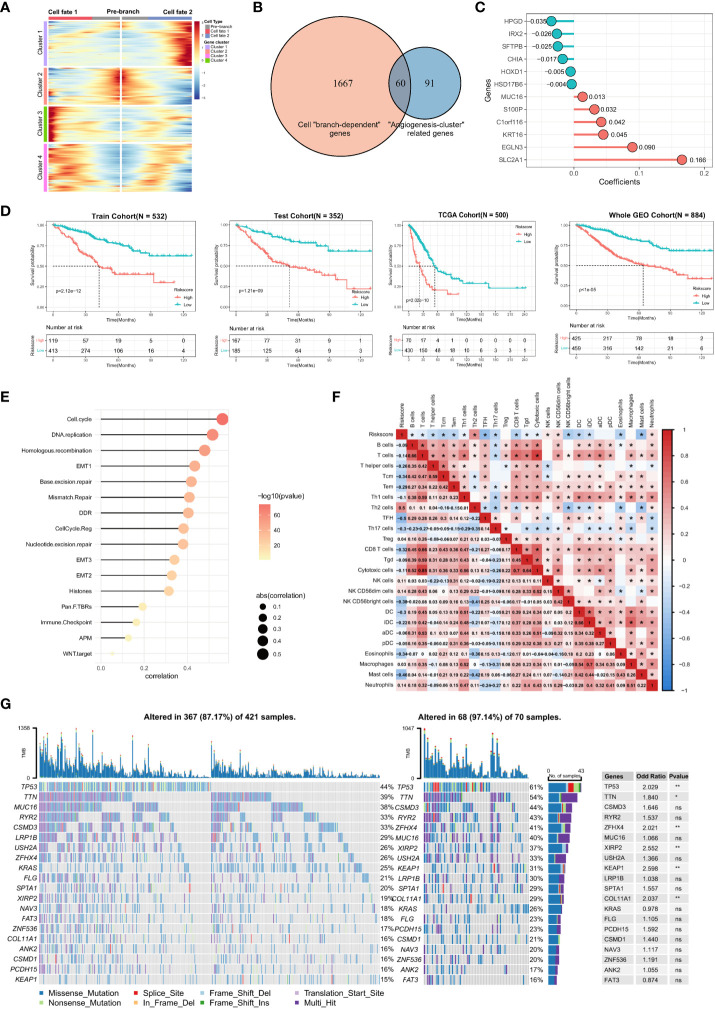
Construction of angiogenic prognostic model and its prognostic value assessment. **(A)** Finding of all genes that differ between the cell branches. The center of the heatmap is the start of the pseudotime, and to the sides are the dynamics of genes associated with different cell fates or branches. The columns in the heatmap are pseudotimes and the rows are genes. The cell state branch-related genes can be clustered into four gene clusters based on co-expression relationships. **(B)** A total of 60 genes were intersected by cell “branch-dependent” genes and “angiogenesis-clusters” related genes. **(C)** Twelve model genes and their coefficients were identified based on univariate Cox regression and Lasso regression analysis. **(D)** Kaplan-Meier curves for overall survival (OS) in the high ARS and low ARS groups were evaluated in the training cohort (N = 532), test cohort (N = 352), external independent validation cohort TCGA cohort (N = 500), and Whole GEO cohort (N = 884), respectively. **(E)** Correlation of ARS with cancer-related biological features and **(F)** the degree of immune cell infiltration using the Spearman analysis. **(G)** Differences in somatic mutations in the tumor genome between the high-ARS and low-ARS groups and statistical tests.

To find all genes that differ between the branches, that is, cell differentiation trajectories, we used the branched expression analysis modeling (BEAM) to find “branch-dependent” genes (**Figure 6A**). These genes are associated with cell differentiation trajectories and also with angiogenic activation. Therefore, we took the intersection of cell branch-related genes and angiogenesis clusters-related genes, which are essential for angiogenic clustering and cell differentiation trajectories in lung adenocarcinoma. Then, to facilitate the assessment of the individualized prognosis of LUAD and guide treatment, a prognostic model, namely the angiogenic risk score (ARS), was developed based on these 60 intersecting genes (**Figure 6B**). The model comprised 12 genes identified via univariate Cox regression and Lasso regression analyses: ARS = Exp(HPGD) * (–0.035) + Exp(IRX2) * (–0.026) + Exp(SFTPB) * (–0.025) + Exp(CHIA) * (–0.017) + Exp(HOXD1) * (–0.005) + Exp(HSD17B6) * (–0.004) + Exp(MUC16) * (0.013) + Exp(S100P) * (0.032) + Exp(C1orf116) * (0.042) + Exp(KRT16) * (0.045) + Exp(EGLN3) * (0.090) + Exp(SLC2A1) * (0.166) (**Figure 6C**). The clinical significance of the prognostic model was assessed, and the low-ARS group had a significant survival benefit with good clinical efficacy for predicting 3-year overall survival in the training set, validation set, TCGA independent validation set, and the whole GEO dataset (**Figure 6D**), with AUC values of 0.71, 0.71, 0.68 and 0.70, respectively (**Supplementary Figure S4A**). Multivariate Cox regression analysis integrating the age, sex, pathological stage, smoking history, and ARSs of patients revealed that ARS was an independent biomarker for the prognosis of LUAD (HR, 3.12; 95% CI, 2.36–4.12; P < 0.001, **Supplementary Figure S4B**).

In addition, a positive correlation was observed between ARS and cancer-related biological signatures reported by Mariathasan et al, especially for cell cycle, EMT, and immune checkpoints, which have been reported to promote proliferation, metastasis, and immune escape in LUAD (**Figure 6E**). These results validate that ARS is associated with a worse prognosis and can be used as an independent prognostic biomarker. Furthermore, the correlation between ARS and immune cell infiltration in the immune microenvironment was analyzed, which revealed that ARS fairly characterized the immune microenvironment. ARS had a positive correlation with Th2 cells (r = 0.5, P < 0.05) and neutrophils (r = 0.14) but a negative correlation with T cells (r = –0.14), Tcm cells (r = –0.34), Tem cells (r = –0.29), CD8 T cells (r = –0.32), TFH cells (r = –0.5), DC (r = -0.3), eosinophils (r = –0.34) and mast cells (r = –0.46) (**Figure 6F**). These results suggest that an increasingly strong tumor-suppressive immune microenvironment is characterized by elevated ARSs. In addition, various immune cells extensively interact with each other, reflecting the complexity of TME.

Furthermore, mutated genes in LUAD were identified in the high- and low-ARS groups. The results showed that both groups had different somatic mutation patterns. The mutation frequency of TP53 (61% versus 44%, respectively; OR, 2.029; P < 0.01), TTN (54% versus 39%, respectively; OR, 1.84; P < 0.05), ZFHX4 (41% versus 26%, respectively; OR, 2.021; P < 0.01), XIRP2 (37% versus 19%, respectively; OR, 2.552; P < 0.01), KEAP1 (31% versus 15%, respectively; OR, 2.598; P < 0.01) and COL11A1 (29% versus 16%, respectively; OR, 2.037; P < 0.01) was higher in the high-ARS group, suggesting that angiogenesis relates to the occurrence of somatic mutations in tumor cells (**Figure 6G**). Therefore, ARS constructed based on angiogenesis-related genes can help to assess TME and genomic somatic mutation patterns in each patient with LUAD, indicating that different ARSs may predict different chemotherapeutic and immunotherapeutic effects.

### 3.7 SLC2A1 promotes angiogenesis in lung adenocarcinoma

**Figure 7:**
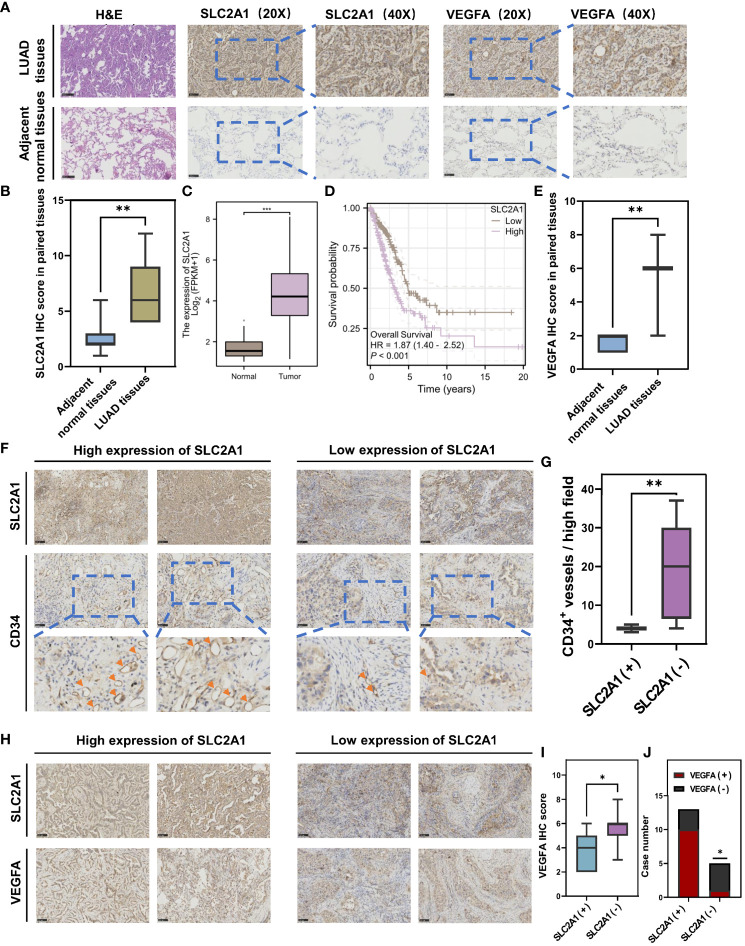
Immunohistochemical staining validates that SLC2A1 promotes angiogenesis in lung adenocarcinoma. **(A)** Immunohistochemical staining of SLC2A1 and VEGFA in lung adenocarcinoma tissues and paracarcinoma tissues. **(B)** The t-test for SLC2A1 IHC score in paired tissues. **(C)** Differential expression of SLC2A1 in lung adenocarcinoma in the TCGA database. **(D)** Overall survival of high and low expression of SLC2A1 in lung adenocarcinoma in the TCGA database. **(E)** Differential expression of VEGFA in lung adenocarcinoma in the TCGA database. **(F)** Immunohistochemical staining of CD34+ microvessels in high and low SLC2A1 expression groups. **(G)** The t-test for the number of CD34+ microvessels per high field in high and low SLC2A1 expression groups. (SLC2A1(+), High SLC2A1 expression group; SLC2A1 (–), Low SLC2A1 expression group). **(H)** Immunohistochemical staining of VEGFA in high and low SLC2A1 expression groups. **(I)** The t-test for VEGFA IHC score in high and low SLC2A1 expression groups. **(J)** Correlation between SLC2A1 and VEGFA by chi-square test.

The ARS prognostic model was established based on the lasso regression algorithm. Among them, SLC2A1 was found to have the largest Lasso regression coefficient of 0.166 and as a high-risk gene, which had the greatest impact on the model and drove us to further validate the role of SLC2A1 on angiogenesis. Consequently, we collected cancerous and paracancerous tissues from seven pairs of lung adenocarcinoma patients and performed immunohistochemical staining for SLC2A1 and VEGFA (**Figure 7A**), and statistical analyses showed that the expression of SLC2A1 and VEGFA was significantly upregulated in lung adenocarcinoma tissues (**Figures 7B, C**), which was in agreement with the expression of SLC2A1 in the TCGA public database (**Figure 7D**). Meanwhile, we found that the expression level of SLC2A1 was significantly associated with the prognosis of lung adenocarcinoma patients, and patients in the high-expression SLC2A1 group had a significantly lower overall survival rate (**Figure 7E**, HR = 1.87, P<0.001). Tumor tissues from 18 patients with lung adenocarcinoma were collected subsequently, and the correlation between SLC2A1 expression level and microvessel density was observed by immunohistochemical staining. Here we visualized the proliferation of microvessels by immunohistochemical staining of CD34. The microvessels in the SLC2A1 high-expression group were shown to be significantly proliferated under high magnification, and the number of CD34-positive microvessels was significantly higher at 22.70 ± 10.34 than that in the low-expression group, which was 4.625 ± 1.506 (Mean ± SD) (**Figure 7F**), and the difference was statistically significant (**Figure 7G**).

Meanwhile, we further verified the role that SLC2A1 mediates VEGFA secretion in lung adenocarcinoma tissues. We examined the expression levels of SLC2A1 and VEGFA in the tumor tissues of 18 lung adenocarcinoma patients by immunohistochemical staining, and the IHC results showed that high expression of SLC2A1 was significantly correlated with the increased secretion of VEGFA (**Figures 7H, I**). The chi-square test showed that more samples in the high-expressing SLC2A1 group overexpressed VEGFA, OR = 13.33, P = 0.0474 (**Figure 7J**), suggesting that patients with high expression of SLC2A1 are more at risk of overexpressing VEGFA, which promotes angiogenesis in tumors.

### 3.8 Prediction of immunotherapeutic and chemotherapeutic effects and construction of an individualized nomogram based on ARS

**Figure 8:**
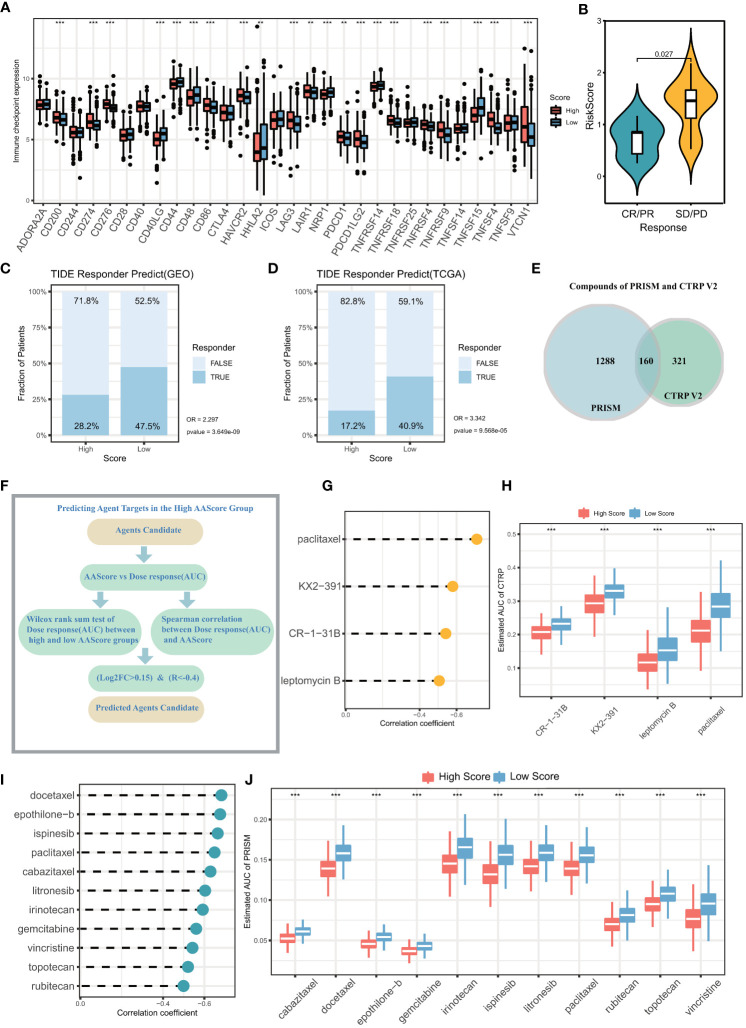
Prediction of immunotherapy effects and sensitive chemotherapeutic agents in the high and low ARS groups. **(A)** Differential expression of immune checkpoints in the high ARS and low ARS groups. **(B)** ARS differences between samples in the group with and without clinical response to immunotherapy. The proportion of immunotherapy with clinical response in the High ARS and Low ARS groups in the **(C)** GEO cohort and **(D)** TCGA cohort was predicted based on the TIDE algorithm. GEO cohort: No immunotherapy response in High ARS versus Low ARS (OR =2.297, p<0.001). TCGA cohort: No immunotherapy response in High ARS versus Low ARS (OR = 3.342, p<0.001). **(E)** Number of chemotherapy drugs in PRISM database and CTRP V2 database. **(F)** Screening of sensitive chemotherapeutic agents based on analysis of variance log2FC and Spearman correlation analysis. **(G)** The correlation between the area under the drug dose-response curve (AUC) and ARS in patients with lung adenocarcinoma was calculated from drug sensitivity data in the PRISM database. **(H)** The difference between the area under the drug dose-response curve (AUC) between the high ARS and low ARS groups was calculated based on the PRISM database. **(I)** The correlation between the area under the drug dose-response curve (AUC) and ARS in patients with lung adenocarcinoma was calculated from drug sensitivity data in the CTRP V2 database. **(J)** The difference between the area under the drug dose-response curve (AUC) between the high ARS and low ARS groups was calculated based on the CTRP V2 database.

In recent years, both immunotherapy and chemotherapy have played an important role in remodeling TME for the treatment of LUAD. The abovementioned results indicate that ARS is associated with the TME of LUAD, somatic mutations in LUAD cells, and the clinical immunotherapeutic and chemotherapeutic effects, suggesting that ARS can facilitate individualized prediction of the efficacy of immunotherapy in patients with LUAD to guide the selection of chemotherapeutic drugs. Furthermore, a majority of immune checkpoints were differently expressed in two groups (**Figure 8A**). High expression of checkpoints is involved in promoting the immune escape of LUAD cells, and these checkpoints mediate the immunosuppressive microenvironment, which may be attributed to the poor prognosis of the high-ARS group. These results suggest that the two groups respond differently to immunotherapy. Furthermore, the SD/PD (Stable disease/Progressive disease) group in the LUAD immunotherapy cohort (GSE126044) had higher ARSs, leading to a poor response to immunotherapy (**Figure 8B**). In addition, the TIDE algorithm was used to assess immunotherapy response in the GEO and TCGA cohorts. The response to immunotherapy was poorer in the high-ARS group than in the low-ARS group, indicating that patients with low ARSs can benefit from ICB treatment (GEO cohort immunotherapy non-response rate: 71.8% versus 52.5%, respectively; OR, 2.297; P < 0.001) (**Figure 8C**), (TCGA cohort immunotherapy non-response rate: 82.8% versus 59.1%; OR, 3.342; P < 0.001) (**Figure 8D**).

Given that ARS significantly affects pathways such as drug metabolism and mediates multiple oncogenic signaling pathways, sensitive chemotherapeutic agents for LUAD can be identified based on ARS. To analyze the potential of ARS as a biomarker for predicting sensitivity to chemotherapeutic agents, the sensitivity of patients with LUAD to chemotherapeutic agents was evaluated based on drug sensitivity data (**Figure 8E**) extracted from the PRISM (1448 compounds) and CTRP V2 (481 compounds) databases. The expression data extracted from CCLE were used as a training cohort. The area under the dose-response curve (AUC) was used to quantify drug sensitivity, with higher AUC values representing lower drug sensitivity. Sensitive drugs were screened using the Wilcoxon test and Spearman correlation analysis (log2FC > 0.15, r < –0.4, **Figure 8F**). Based on the CTRP V2 database, 4 chemotherapeutic agents were identified, including paclitaxel, KX2-391, CR-1-31B, and leptomycin (**Figures 8G, H**). In addition, 11 chemotherapeutic drugs with high sensitivity were identified based on the PRISM database using the same screening criteria, including docetaxel, epothilone-b, ispinesib, paclitaxel, cabazitaxel, litronesib, irinotecan gemcitabine, vincristine, topotecan, and rubitecan (**Figures 8I, J**). Patients with high ARSs may benefit from the above mentioned chemotherapeutic agents.

**Figure 9:**
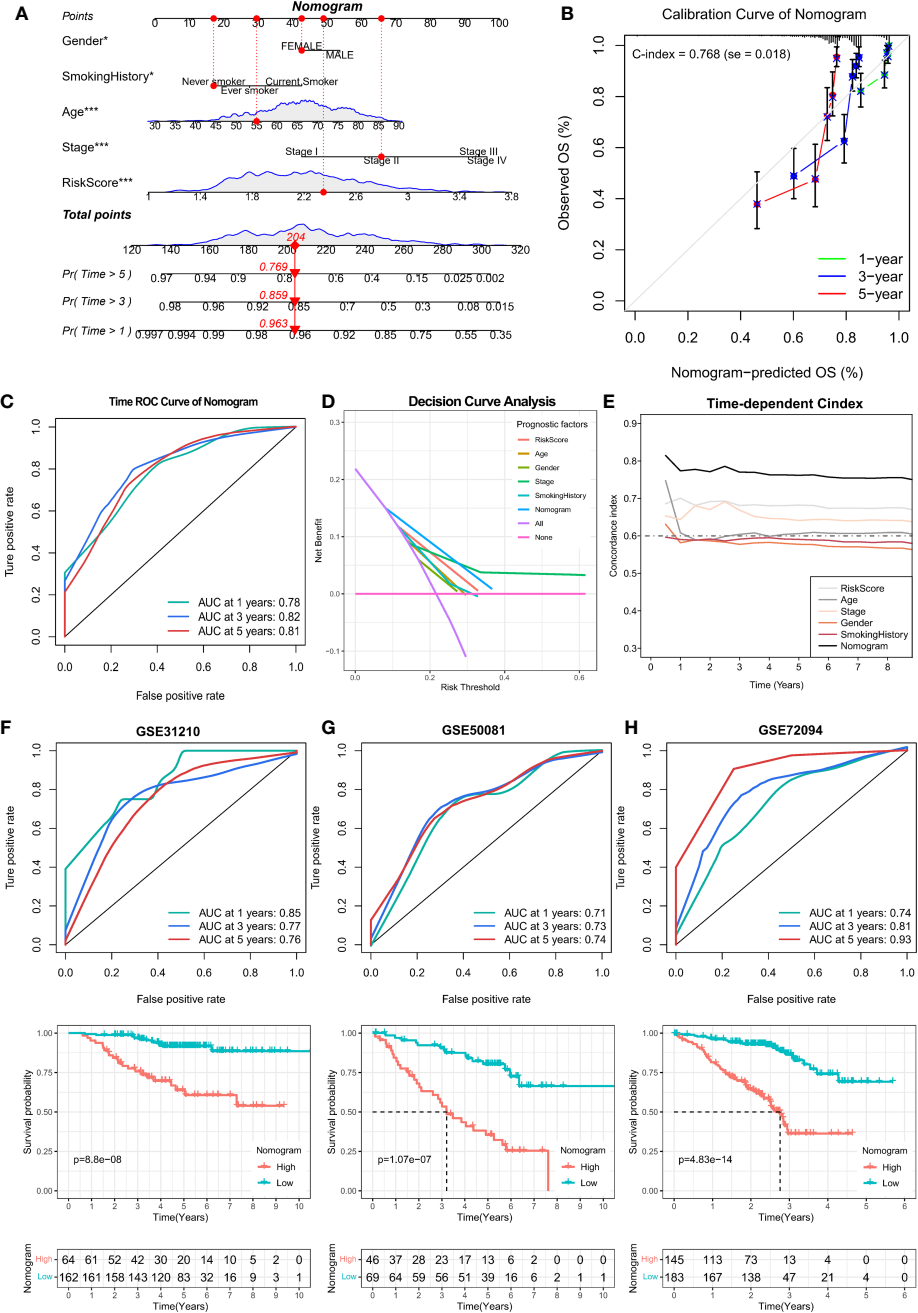
Prognostic value analysis of Nomogram was constructed by combining age, gender, pathological stage, smoking history, and ARS. **(A)** Construction of Nomogram with 1-, 3- and 5-year survival rates of 0.963, 0.859, and 0.769 for the example sample, respectively. **(B)** Calibration curve to assess the prediction accuracy of Nomogram with a Concordance index (C-index) of 0.768 (se = 0.018). **(C)** The ROC curves of the Nomogram assessed their 1-, 3-, and 5-year overall survival with AUC values of 0.78, 0.82, and 0.81, respectively. **(D)** Decision curve analysis as well as **(E)**Time-dependent C-index calculations showed that the Nomogram outperformed any other clinical characteristics in predicting overall survival. **(F-H)** Kaplan-Meier and ROC curves for overall survival for the GSE31210, GSE50081, and GSE72094 cohorts.

Furthermore, the independent prognostic marker ARS was combined with clinical prognostic characteristics such as age, gender, pathological stage, and smoking history to construct a nomogram for clinical prognostic prediction (**Figure 9A**), which can better assess the risk factors and guide subsequent treatment strategies. The calibration curve of the nomogram showed good performance with a concordance index (C-index) of 0.768 (**Figure 9B**), and the AUC of the ROC curve for predicting 1-, 3- and 5-year survival were 0.78, 0.82, and 0.81, respectively (**Figure 9C**), indicating that the nomogram had good accuracy in predicting overall survival. Decision curve analysis (DCA) and time-dependent C-index revealed that the clinical prediction accuracy of the nomogram was superior to that of other clinicopathological features (**Figures 9D, E**), indicating that the nomogram can be used in clinical settings in the future. In addition, we validate the accuracy of the Nomogram in three independent datasets. High and low Nomogram scores showed significant differences, and notably, the AUCs of 5-year overall survival for the Nomogram were 0.76, 0.74, and 0.93, respectively (**Figures 9F-H**), further confirming the clinical predictive performance of Nomogram. In conclusion, the assessment of angiogenesis and the rest of the clinicopathological features can be integrated to assess the prognosis of lung adenocarcinoma patients with great accuracy.

## 4 Discussion

LUAD is a highly heterogeneous malignancy, and several studies have used single-cell and bulk sequencing studies to discuss the heterogeneity of the TME of LUAD (22). Angiogenesis plays a crucial role in promoting tumor growth and metastasis, and vascular endothelial growth factor (VEGF) and inflammatory chemokines exert immunomodulatory effects, which enhance angiogenesis while leading to immunosuppression (23). Studies have indicated the importance of angiogenesis for the differentiation of TME phenotypes (9). Clinically, anti-angiogenic drugs that block VEGF/VEGFR signaling have been successful in treating LUAD; however, they can induce hypoxia, leading to drug resistance, thereby exacerbating immunosuppression and increasing immune checkpoint PD-L1 expression (24). Therefore, an in-depth understanding of angiogenesis and TME interactions can help guide combination therapy for LUAD. Meanwhile, it is crucial to construct prognostic models based on angiogenesis to individually assess the prognosis and microenvironmental status of patients.

In this study, two angiogenic clusters showed different tumor microenvironmental phenotypes and prognostic features. LUAD microenvironment has been categorized into three phenotypes, namely, “inflamed”, “immune-desert”, and “immune-excluded”, which mediate different prognoses and immunotherapeutic responses (25). The inflamed phenotype demonstrates anti-cancer immune activation and has a better prognosis. However, angiogenic Cluster1 in this study was associated with a poor prognosis, demonstrating the characteristics of the immune-deserted and immune-excluded phenotypes, which are characterized by differential activation of oncogenic signaling pathways such as glycolysis, cell cycle, hypoxia, and epithelial–mesenchymal transition. Moreover, immune cell infiltration and the expression of immune-related regulatory factors were downregulated in Cluster1. Angiogenesis mediates different tumor microenvironmental phenotypes in other solid tumors as well (9, 26).

scRNA-seq allows the analysis of interactions between cell subpopulations and specific transcriptional regulators at a high resolution (27). In this study, significant differences were observed in angiogenic activity among different cell types, which validated the heterogeneity of angiogenesis. The highest angiogenic activity was observed in malignant cells, fibroblasts, and neutrophils, which is consistent with the results of previous studies. Unterleuthner et al. demonstrated that cancer-associated fibroblasts (CAFs) promote angiogenesis through the expression of WNT2 (28). Neutrophils have also been reported to secrete pro-angiogenic factors and drive immunosuppression to promote tumor growth (29).

In this study, angiogenic activation was significantly heterogeneous in the malignant cell subpopulation of LUAD; however, the underlying causes and biological mechanisms warrant further investigation. Pseudotime trajectory analysis of malignant LUAD cells revealed the presence of three main cell differentiation states. Furthermore, angiogenesis activated the three cell states with specific transcription factors (regulons). Evaluation of RAS revealed differences in transcription factors regulating the heterogeneity of angiogenic activation in malignant LUAD cells. Transcription factors of State2 cells were found to be associated with angiogenic activation. However, transcription factors of State3 cells mediated lower angiogenic activation, and angiogenic activation was more complex in State 1 cells than in State2 and State3 cells. Altogether, exploring the specific regulon of different cell states is crucial for a deeper understanding of the differences in angiogenic activation in LUAD.

The complex cellular communication in TME drives cancer progression and response to the available therapies (30). In this study, different cell states, that is, different activation states of angiogenic pathways, communicated significantly differently with cells in the TME of LUAD, which further reveals the role of angiogenesis in the crosstalk in TME. Furthermore, multiple ligand–receptor pairs associated with malignant, immune, and stromal cells were identified, some of which have been reported to play a significant role in lung cancer. For example, the TNFRSF12A/Fn14 signaling axis activates NF-κB to promote the survival of LUAD cells (31), and IGF2 promotes neovascularisation in LUAD (32). However, SEMA3B attenuates tumorigenesis and angiogenesis (33). Furthermore, a complex relationship was observed between State1 and angiogenesis, and several novel cellular communication modes of State1 cells were identified. State1 cells were found to communicate closely with fibroblasts and M2-type macrophages via the POSTIN–(ITGAV+ITGB5) and MDK–(ITGA6+ITGB1) signaling pathways, respectively; however, State2 cells promoted tumorigenesis by interacting with microenvironmental cells through a different communication mode, such as the HBEGF–EGFR pathway that induces the proliferation and growth of lung cancer cells (34). State3 cells were also regulated by different ligand–receptor pairs. Therefore, angiogenesis mediates intercellular communication in the LUAD microenvironment.

Previous studies have demonstrated that abnormal angiogenesis is associated with the function and migration of immune cells (35). However, anti-angiogenic therapy has been shown to improve the response to immunotherapy while preventing tumor immune escape (36). Given the significant role of angiogenesis in the prognosis of LUAD and TME, an individualized prognostic model (ARS) based on angiogenesis-related genes was constructed in this study for assessing the TME and survival of patients with LUAD. ARS can be considered an independent prognostic factor for LUAD and can guide individualized treatment strategies. It was significantly correlated with immune-related pathways, cell cycle, and drug metabolism and was significantly positively correlated with the infiltration of Th2 cells and neutrophils. Th2 cells can form an immunosuppressive microenvironment and promote tumor immune escape (37). However, ARS had a significant negative correlation with the infiltration of anti-tumor immune cells such as CD8+ T cells, with the high and low ARSs characterizing the immunosuppressive and anti-tumor immune microenvironments, respectively. Significant differences were observed in mutation frequencies between the high- and low-ARS groups. TP53 mutations significantly increased the expression of immune checkpoints and were associated with the significant clinical benefits of PD-1 inhibitors (38). KEAP1-driven co-mutations in LUAD are closely associated with having high TMB but not responding to immunotherapy (39). In this study, significant differences in mutation frequencies between the high- and low-ARS groups and their close correlation with immunotherapy response indicated that ARS can help to individually assess the immune infiltration status, immunotherapeutic response, and chemotherapeutic drug sensitivity in patients with LUAD. In addition, both immunotherapy cohort and TIDE algorithm predictions suggested that the low-ARS group benefitted from immunotherapy.

Specific sensitive chemotherapeutic agents were predicted in the high-ARS group to guide LUAD chemotherapy. Paclitaxel and docetaxel have been extensively used in the treatment of LUAD (40, 41). Cabazitaxel, paclitaxel (42), and epothilone (43) are commonly used in chemotherapy for advanced non-small cell lung cancer; they stabilize microtubules and cause apoptosis of tumor cells. KX2-391 can reduce cell proliferation and angiogenesis, thereby inhibiting tumor growth (44). Also, gefitinib has excellent efficacy in the treatment of LUAD (45). Irinotecan in combination with gemcitabine and cisplatin can be used as a first-line treatment for advanced LUAD (46). However, the role of CR-1-31B, litronesib, and ispinesib in LUAD remains unclear. Although topotecan, vincristine, and rubitecan are widely used for the treatment of small cell lung cancer, their efficacy in LUAD treatment warrants further investigation. In this study, drug sensitivity analysis revealed that the high-ARS group was more sensitive to the abovementioned drugs, indicating that patients with high ARSs may benefit from these chemotherapeutic drugs.

Given that ARS has a good prognostic value, a multifactorial regression model was constructed, and the accuracy of prognostic prediction (3-year AUC of 0.82) was significantly improved with excellent discrimination (47). The accuracy is comparable to our previously established prognostic models related to sumoylation and M2 macrophages, and ARS can be combined with them in prognostic assessments (48, 49). Although the role of angiogenesis in mediating intercellular crosstalk in the TME of LUAD was examined by analyzing angiogenic pathway activation, the underlying mechanisms warrant comprehensive and in-depth investigation. Therefore, more single-cell sequencing studies should be conducted to refine the exploration of the role of angiogenesis in mediating the TME of LUAD. However, alterations in circRNA and miRNA levels are also important mechanisms (50). Due to the lack of these data, our multi-omics analysis was limited to the mRNA level, and in the future, more abundant and comprehensive data for multi-omics analysis will be needed for further analysis. Finally, the predictive efficiency of the prognostic model established in this study was high in both training and validation cohorts; however, more LUAD and immunotherapy cohorts are required to validate the results to further improve the accuracy of the prognostic model.

## 5 Conclusions

In conclusion, the assessment of angiogenic clusters helps to determine the prognostic and TME characteristics of LUAD. Heterogeneity in the activation of angiogenesis in LUAD is regulated by regulon submodules. There are significant differences in the cell communication patterns in TME between different angiogenic activation states. We further constructed a highly accurate prognostic model to assist in the clinical assessment of individualized LUAD patient prognosis and tumor microenvironment and to facilitate the assessment of immunotherapy response and sensitive chemotherapeutic agents.

## Data availability statement

The datasets presented in this study can be found in online repositories. The names of the repository/repositories and accession number(s) can be found in the article/**Supplementary Material.**

## Ethics statement

The studies involving humans were approved by Medical Ethics Committee at The Affiliated First Hospital of Soochow University. The studies were conducted in accordance with the local legislation and institutional requirements. Written informed consent for participation was not required from the participants or the participants’ legal guardians/next of kin in accordance with the national legislation and institutional requirements.

## Author contributions

LT: Conceptualization, Data curation, Formal analysis, Software, Validation, Visualization, Writing – original draft, Writing – review & editing. ZC: Conceptualization, Formal analysis, Investigation, Methodology, Software, Validation, Visualization, Writing – review & editing, Writing – original draft. JY: Validation, Visualization, Writing – review & editing. QL: Data curation, Methodology, Writing – review & editing. SW: Data curation, Formal analysis, Writing – review & editing. TM: Data curation, Formal analysis, Writing – review & editing. WZ: Data curation, Formal analysis, Software, Validation, Writing – review & editing. HD: Formal analysis, Methodology, Software, Supervision, Validation, Writing – review & editing. SP: Conceptualization, Funding acquisition, Supervision, Writing – review & editing.

## Supplementary material

Supplementary Figure 1 Consensus clustering of lung adenocarcinoma based on angiogenesis-related genes**. (A-C)** Consensus clustering of lung adenocarcinoma samples from the GEO cohort based on the expression of angiogenesis-related genes (K = 2, K values determined from CDF curves). **(D-G)** Survival analysis of clustered results in an independent data set was performed to verify prognostic significance. **(H-I)** Consensus clustering of TCGA cohort based on angiogenesis-related genes (K = 2, K values determined from CDF curves).

Supplementary Figure 2 Cells communicate in the tumor microenvironment with different ligand-receptor pairs. **(A)** Afferent signaling coordination modes of cell-ligand receptor pairs can be divided into two types. **(B)** State1 cells communicate with M2 macrophages via TNFSF12-TNFRSF12A and **(C)** IGF2-(ITGA6+ITGB4). **(D)** State1 cells communicate with M2 macrophages and CD4 T cells via MDK-(ITGA6+ITGB1). **(E)**State1 cells communicate extensively with other cells of the tumor microenvironment via SEMA3B-(NRP2+PLXNA2). **(F)**State1 cells are in close contact with Fibroblasts and Endothelial via POSTN-(ITGAV+ITGB5).

Supplementary Figure 3 Ligand receptor pairs mediating cell communication between cell state3 and the tumor microenvironment. **(A)** State3 cells communicate with Endothelial via CALCA-CALCRL, **(B)** with Fibroblasts via FGF8-FGFR1, and **(C)** with M2-type macrophages and cDC via CGA-FSHR. **(D)** State3 cells send signals to M2-type macrophages and various other cells via ARTN-GFRA1. Through **(E)** LIF-(LIFR+IL6ST) and **(F)**VEGFA-VEGFR1, State3 can communicate extensively with Mast, M2-type macrophages, and Endothelial each other.

Supplementary Figure 4 Prognostic performance assessment for ARS risk scores. **(A)** Receiver operating characteristic (ROC) curves for overall survival (OS) in the high ARS and low ARS groups were evaluated in the training cohort (N = 532), test cohort (N = 352), external independent validation cohort TCGA cohort (N = 500), and Whole GEO cohort (N = 884), respectively. **(B)** Multivariate Cox analysis combining age, sex, pathological stages, smoking history, and other clinical characteristics confirmed the independent prognostic value of ARS in lung adenocarcinoma (HR, 3.12 (95% CI, 2.36-4.12), P<0.001).
