# Supplementary figures and images for: Single-cell and Bulk RNA-Seq reveal angiogenic heterogeneity and microenvironmental features to evaluate prognosis and therapeutic response in lung adenocarcinoma
Source: Front Immunol. 2024 Feb 8;15:1352893. doi: 10.3389/fimmu.2024.1352893 (PMC10882092; doi:10.3389/fimmu.2024.1352893)

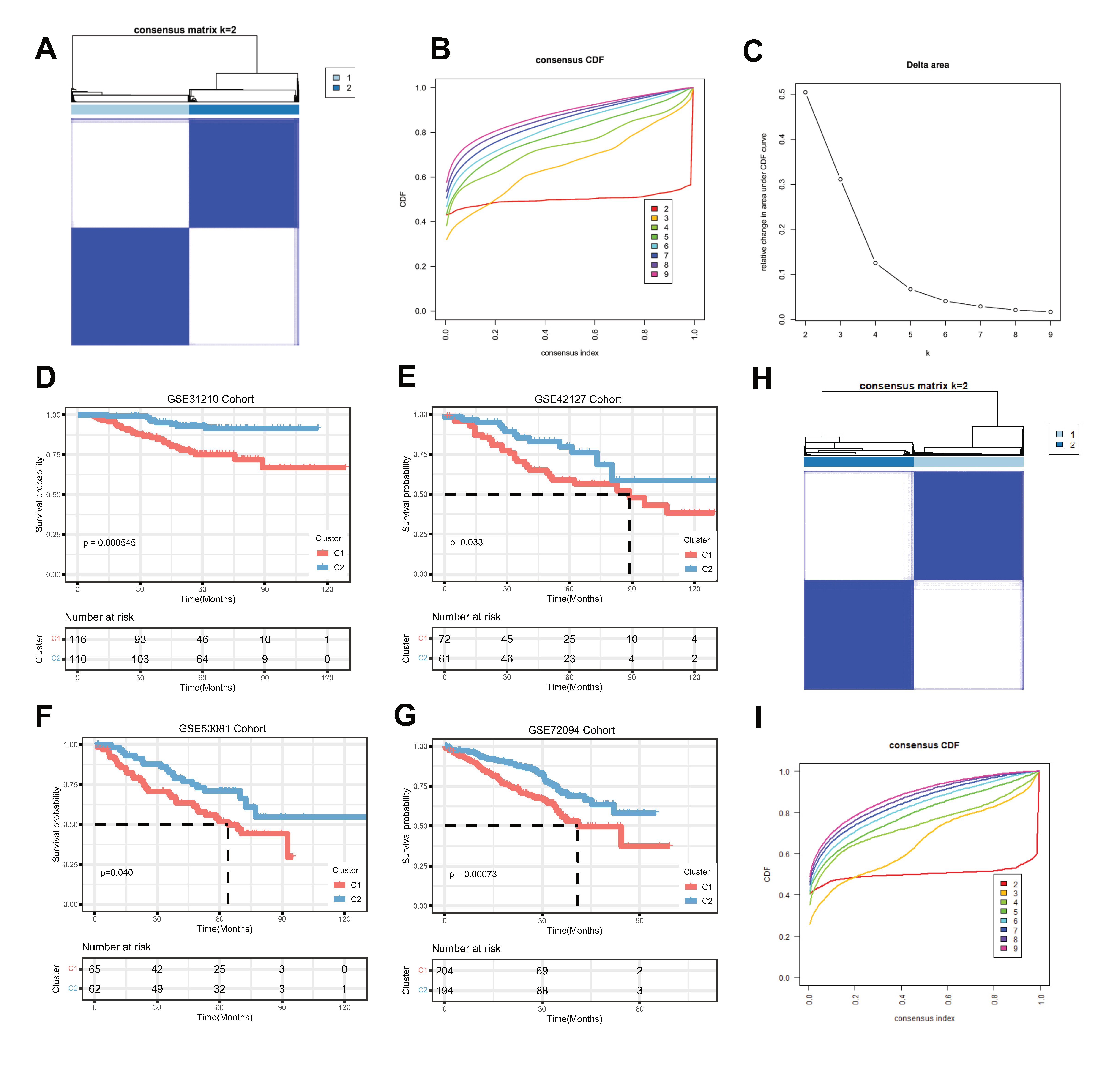

Supplement: Supplementary Figure 1 — Consensus clustering of lung adenocarcinoma based on angiogenesis-related genes. (A-C) Consensus clustering of lung adenocarcinoma samples from the GEO cohort based on the expression of angiogenesis-related genes (K = 2, K values determined from CDF curves). (D-G) Survival analysis of clustered results in an independent data set was performed to verify prognostic significance. (H-I) Consensus clustering of TCGA cohort based on angiogenesis-related genes (K = 2, K values determined from CDF curves). [file Image_1.tif]

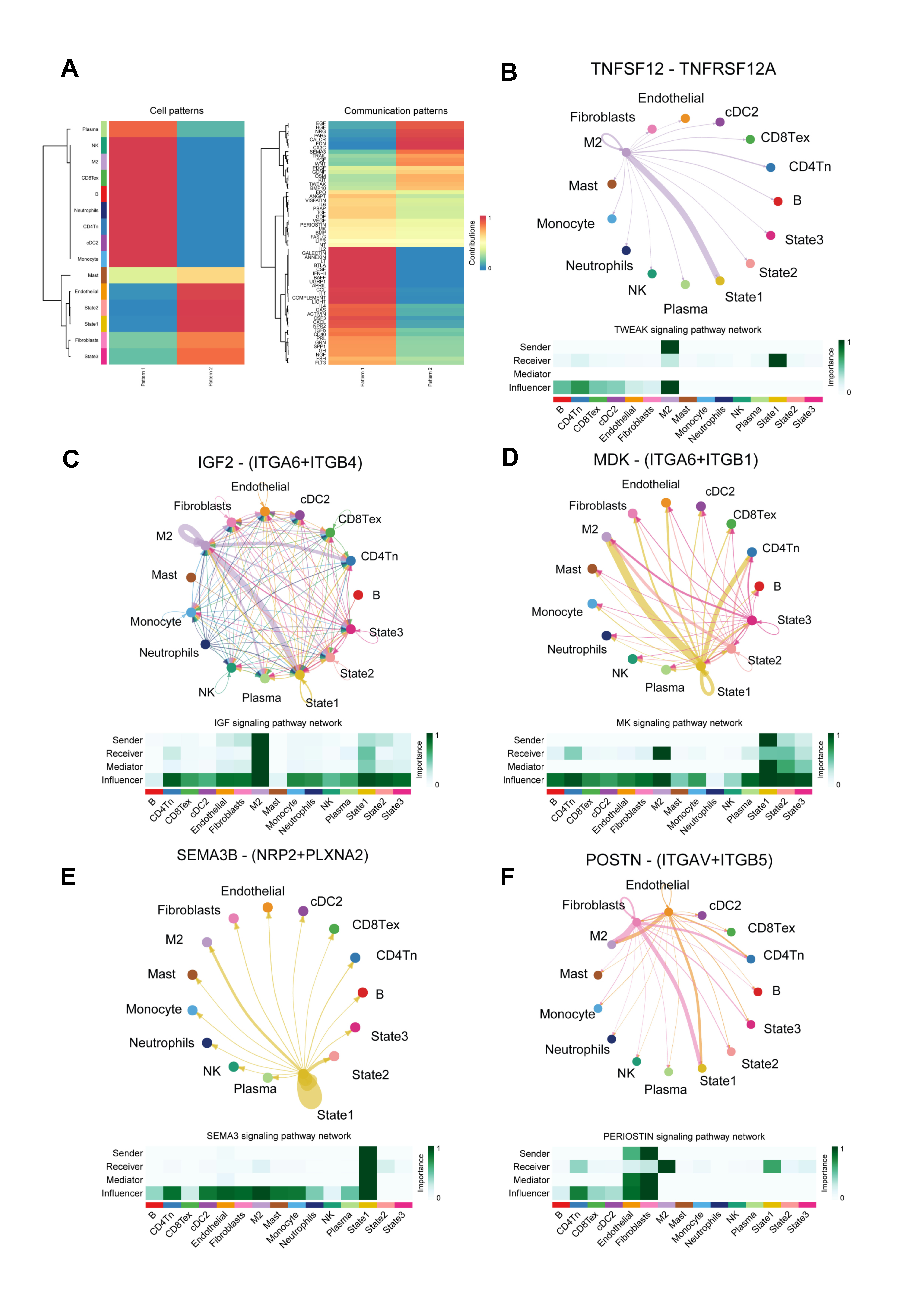

Supplement: Supplementary Figure 2 — Cells communicate in the tumor microenvironment with different ligand-receptor pairs. (A) Afferent signaling coordination modes of cell-ligand receptor pairs can be divided into two types. (B) State1 cells communicate with M2 macrophages via TNFSF12-TNFRSF12A and (C) IGF2-(ITGA6+ITGB4). (D) State1 cells communicate with M2 macrophages and CD4 T cells via MDK-(ITGA6+ITGB1). (E)State1 cells communicate extensively with other cells of the tumor microenvironment via SEMA3B-(NRP2+PLXNA2). (F)State1 cells are in close contact with Fibroblasts and Endothelial via POSTN-(ITGAV+ITGB5). [file Image_2.tif]

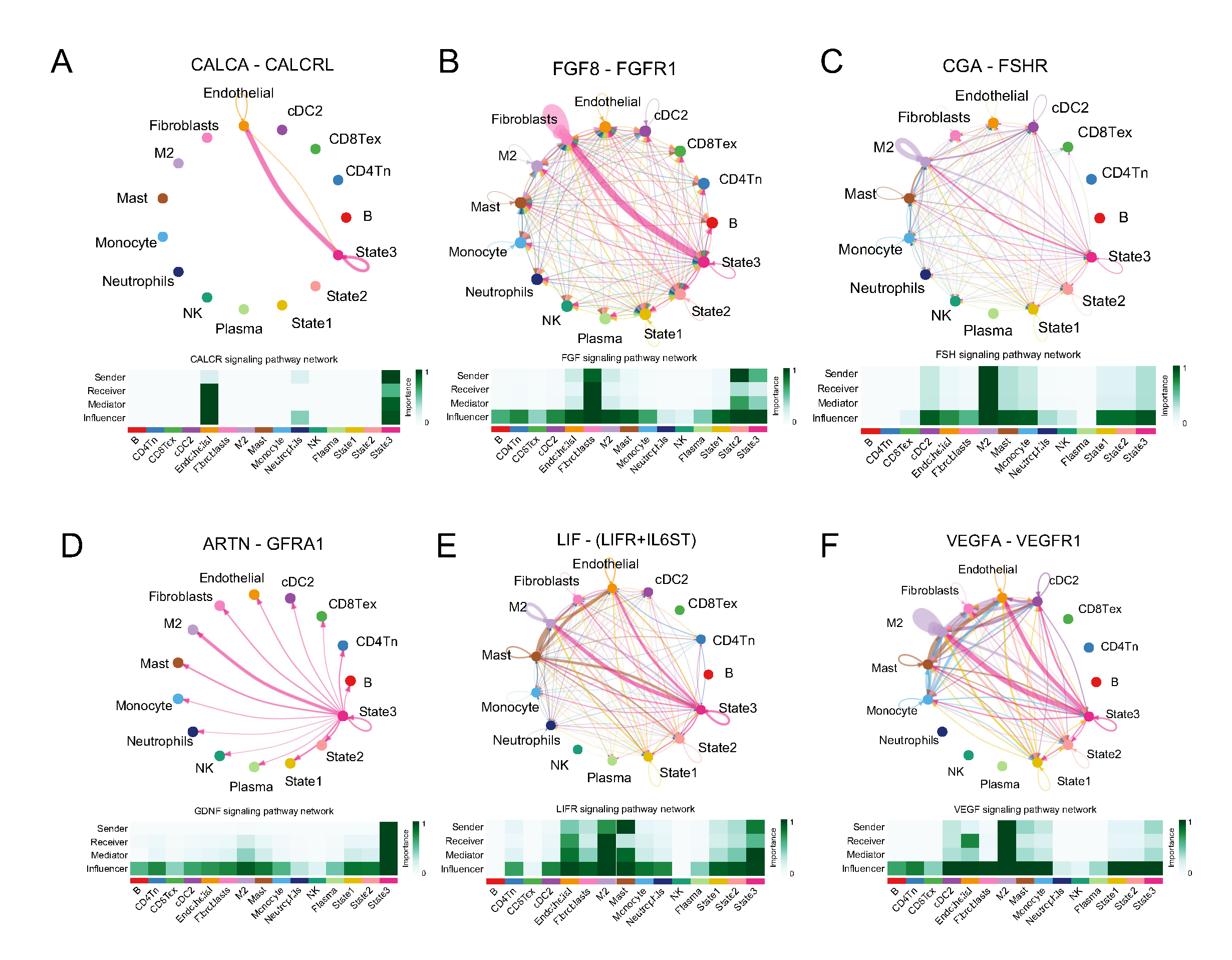

Supplement: Supplementary Figure 3 — Ligand receptor pairs mediating cell communication between cell state3 and the tumor microenvironment. (A) State3 cells communicate with Endothelial via CALCA-CALCRL, (B) with Fibroblasts via FGF8-FGFR1, and (C) with M2-type macrophages and cDC via CGA-FSHR. (D) State3 cells send signals to M2-type macrophages and various other cells via ARTN-GFRA1. Through (E) LIF-(LIFR+IL6ST) and (F)VEGFA-VEGFR1, State3 can communicate extensively with Mast, M2-type macrophages, and Endothelial each other. [file Image_3.tif]

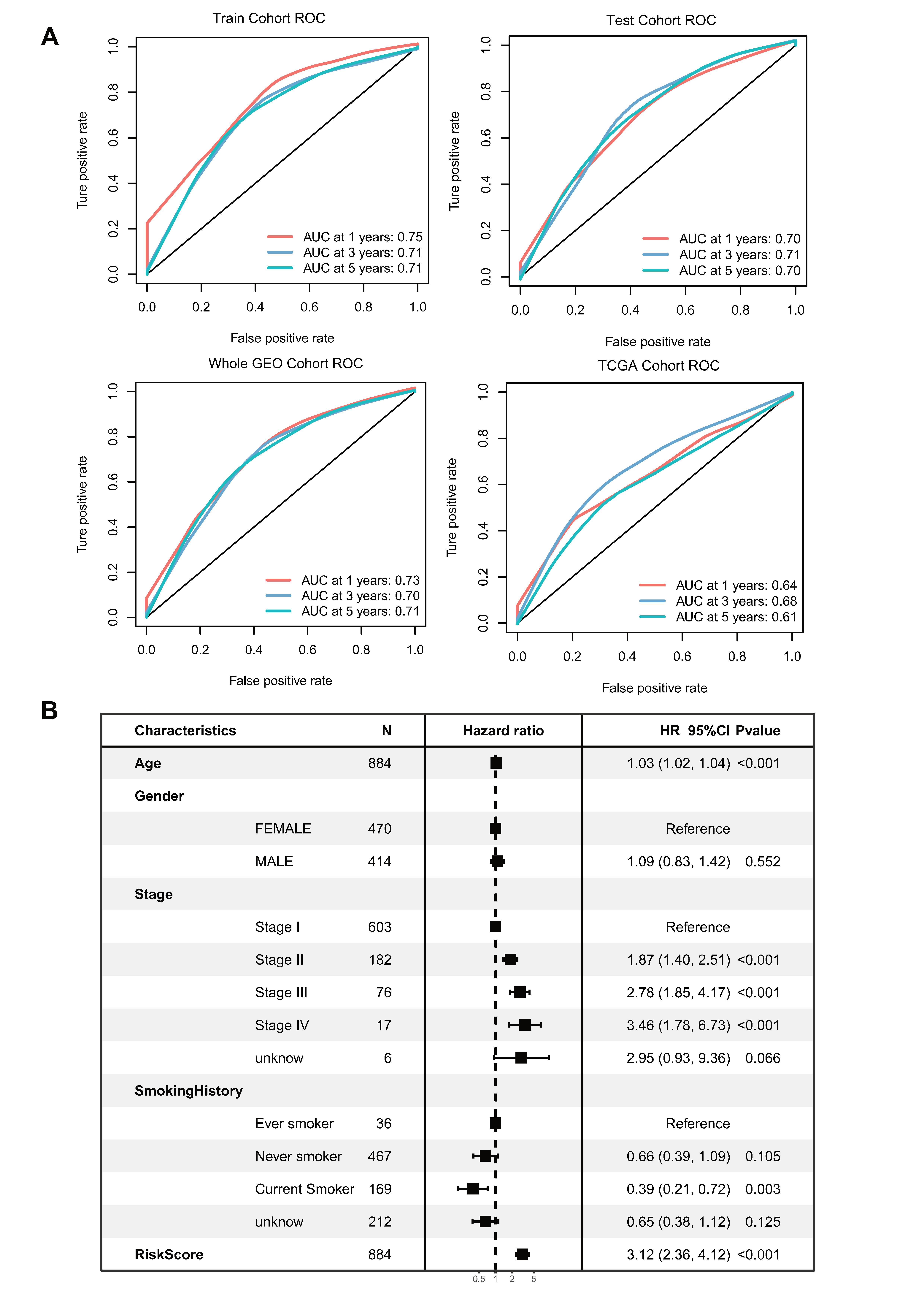

Supplement: Supplementary Figure 4 — Prognostic performance assessment for ARS risk scores. (A) Receiver operating characteristic (ROC) curves for overall survival (OS) in the high ARS and low ARS groups were evaluated in the training cohort (N = 532), test cohort (N = 352), external independent validation cohort TCGA cohort (N = 500), and Whole GEO cohort (N = 884), respectively. (B) Multivariate Cox analysis combining age, sex, pathological stages, smoking history, and other clinical characteristics confirmed the independent prognostic value of ARS in lung adenocarcinoma (HR, 3.12 (95% CI, 2.36-4.12), P<0.001). [file Image_4.tif]
